# Supplementary material for: Palmitoylated Importin α Regulates Mitotic Spindle Orientation Through Interaction with NuMA
Source: bioRxiv. 2024 Oct 25:2024.10.25.620315. Preprint. [Version 1] doi: 10.1101/2024.10.25.620315 (PMC11527331; doi:10.1101/2024.10.25.620315)
Supplement: Supplementary file 1 [file media-1.docx]

**Supplemental Figures**

**Supplemental Figure 1. Proteome Screens Confirm Palmitoylation of Human Importin** **α****-1 and Enrichment of NLS Containing Proteins by Cellular Localization and Function.**

A) Prediction of palmitoylated cysteine residues in Human Importin α-1 (KPNA2) by GPS-Palm. Prediction score is on a 0-1 scale with 0 representing low confidence of palmitoylation and 1 representing high confidence of palmitoylation. Three highest confidence residues are highlighted in green. 

B) Cellular localization of NLS containing proteins not predicted to be in the nucleus.

C) Cellular functions of NLS containing proteins found at the PM.

**Supplemental Figure 2. KPNA2 Cellular Localization Boundary Determination.**

Representative immunofluorescence and bright-field images for cells used in Figure 1A to determine KPNA2 localization and cell boundary determination for each drug treatment. Scale bar=5μm.

**Supplemental Figure 3. CaaX Modified Importin** **α** **Localizes to the Plasma Membrane Independent of Palmitoylation.**

Fluorescent images of HCT116 cells transfected with importin α-mCherry, mCherry-CaaX or importin α-mCherry-CaaX treated with DMSO, Wnt-C59 or palmostatin. Importin α-mCherry-CaaX localizes to the plasma membrane in all drug treatments. Scale bar=5μm.

**Supplemental Figure 4. Wnt-C59 Treated** ***Xenopus laevis*** **Tadpoles Exhibit Shortened Tail Length.**

A) NF Stage 42 *X. laevis* tadpole tails following 48 hours of DMSO treatment

B) NF Stage 42 *X. laevis* tadpole tails following 48 hours of Wnt-C59 treatment

**Supplemental Figure 5. Importin** **α** **Overexpression Produces Severe Developmental Defects in** ***Xenopus laevis*** **Embryos.**

Fluorescent images of *X. laevis* embryos co-injected with importin α-mCherry-CaaX pcDNA4TO and pcDNA6TR at 24, 48, and 72 hours post fertilization.  Embryos were injected at either the 1 cell, 2 cell (injected into 1 of 2 cells), or 16 cell stage (injected into the D11 blastomere).  All 1 and 2 cell injected embryos exhibit high mortality and severe developmental defects. D11 injected embryos had better survivability though some embryos still exhibited defects.
